# Cellular Senescence as the Causal Nexus of Aging

**DOI:** 10.3389/fgene.2016.00013

**Published:** 2016-02-12

**Authors:** Naina Bhatia-Dey, Riya R. Kanherkar, Susan E. Stair, Evgeny O. Makarev, Antonei B. Csoka

**Affiliations:** ^1^Epigenetics Laboratory, Department of Anatomy, Howard UniversityWashington, DC, USA; ^2^Vision Genomics, LLCWashington, DC, USA; ^3^InSilico Medicine, Emerging Technology Center, Johns Hopkins UniversityBaltimore, MD, USA

**Keywords:** aging, senescence, proliferation, causal nexus, august weismann, germline, mortality, immortality

## Abstract

In this paper we present cellular senescence as the ultimate driver of the aging process, as a “causal nexus” that bridges microscopic subcellular damage with the phenotypic, macroscopic effect of aging. It is important to understand how the various types of subcellular damage correlated with the aging process lead to the larger, visible effects of anatomical aging. While it has always been assumed that subcellular damage (cause) results in macroscopic aging (effect), the bridging link between the two has been hard to define. Here, we propose that this bridge, which we term the “causal nexus”, is in fact cellular senescence. The subcellular damage itself does not *directly* cause the visible signs of aging, but rather, as the damage accumulates and reaches a critical mass, cells cease to proliferate and acquire the deleterious “senescence-associated secretory phenotype” (SASP) which then leads to the macroscopic consequences of tissue breakdown to create the physiologically aged phenotype. Thus senescence is a precondition for anatomical aging, and this explains why aging is a gradual process that remains largely invisible during most of its progression. The subcellular damage includes shortening of telomeres, damage to mitochondria, aneuploidy, and DNA double-strand breaks triggered by various genetic, epigenetic, and environmental factors. Damage pathways acting in isolation or in concert converge at the causal nexus of cellular senescence. In each species some types of damage can be more causative than in others and operate at a variable pace; for example, telomere erosion appears to be a primary cause in human cells, whereas activation of tumor suppressor genes is more causative in rodents. Such species-specific mechanisms indicate that despite different initial causes, most of aging is traced to a single convergent causal nexus: senescence. The exception is in some invertebrate species that escape senescence, and in non-dividing cells such as neurons, where senescence still occurs, but results in the SASP rather than loss of proliferation plus SASP. Aging currently remains an inevitable endpoint for most biological organisms, but the field of cellular senescence is primed for a renaissance and as our understanding of aging is refined, strategies capable of decelerating the aging process will emerge.

## Introduction

Until recently the biology of aging may have appeared to be hopelessly complex, but we can nevertheless apply a strictly rational approach toward solving the problem: what is the overarching, fundamental basis of biological aging? There *must* be a foundational reason for aging that leads to the effects that we observe at the macroscopic, organismal level.

**FIGURE 1:**
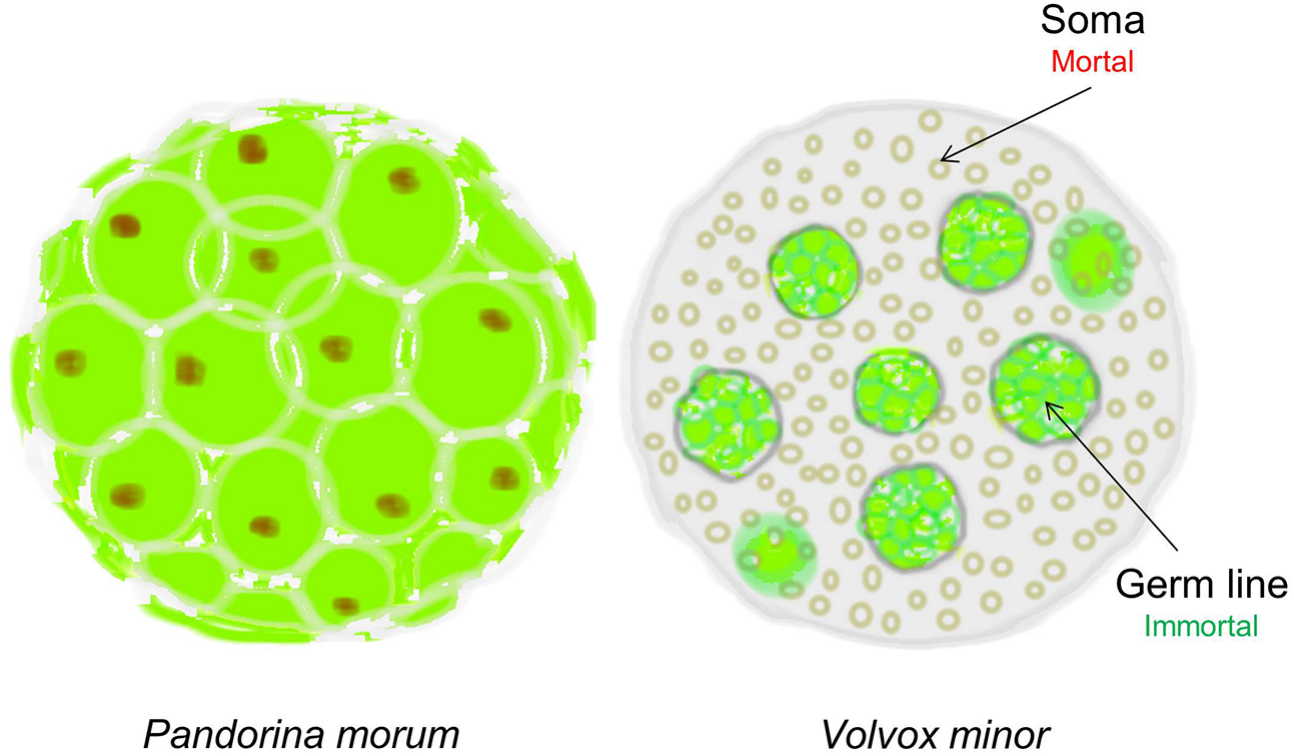
**The evolution of cellular diversity as the origin of cellular senescence.** *Pandorina morum* on the left, which is immortal, has a single cell type; whereas the mortal *Volvox minor* on the right has two distinct cell types representing the immortal germ line and the mortal soma in the same multicellular organism. Only the immortal germline cells in *Volvox* can produce the next generation (Figure adapted from West, 2003).

In 1881 the evolutionary biologist August Weismann took such a rational approach and proposed that “Death takes place because a worn-out tissue cannot forever renew itself, and because a capacity for increase by means of cell division is not everlasting but finite.” How did he arrive at such a bold conclusion? Weismann observed that during evolution, simple multicellular organisms such as Pandorina Morum, which were immortal, gradually evolved into mortal organisms such as Volvox Minor (West, 2003). The absolutely crucial difference between these two organisms is that while Pandorina’s cells were undifferentiated and divided without limit, Volvox’s cells had differentiated into two very different types: the Soma (body) cells, and the Germ (reproductive) cells (**Figure 1**). Thus, while the germ line has retained the capacity for infinite renewal, the body cells (soma) have not; they age and expire.

**FIGURE 2:**
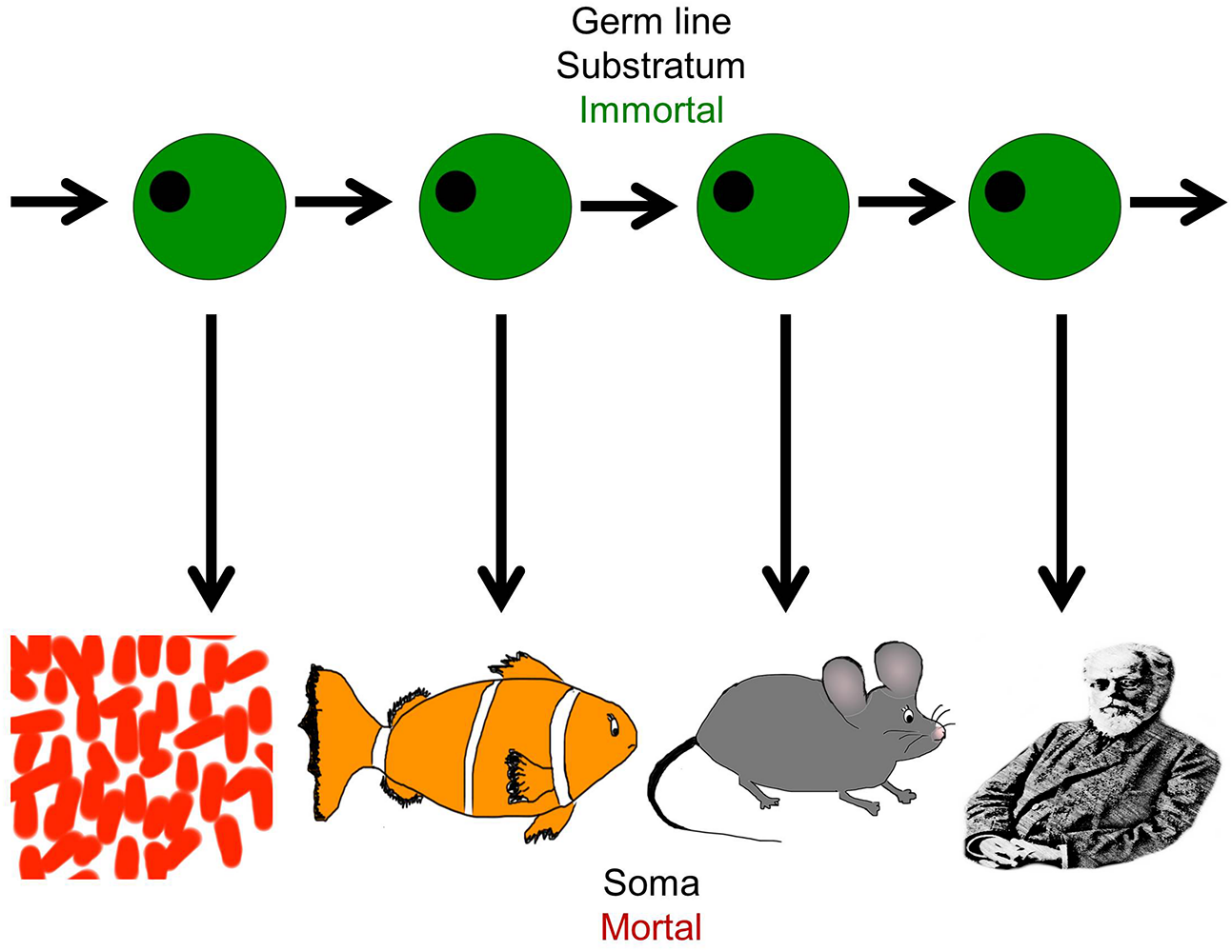
**The dichotomy of germ line and soma: organismal diversity.** The figure represents the evolution of complex organisms from simpler forms through the immortal germline. Each species is capable of self-propagation *only* through the germ line, and this has evolved and given rise to increasingly complex organisms from the simple organism like *Volvox* to the most complex such as *Homo sapiens*. It represents the co-existence of cellular immortality and mortality in the same organism (Figure adapted from West, 2003).

Life on earth has been perpetuated for billions of years throughout evolution, sustained by imperishable germ line cells, but individual organisms perish after each generation because somatic cells cannot divide indefinitely (**Figure 2**). Closer analysis reveals that in biological systems, the variability in proliferative capacity of different types of somatic cells from different species is exhibited over a wide range, from very limited to extensive. But nevertheless, the immortality inherent in the earliest unicellular organisms evolved into complex, *mortal* multicellular organisms that acquired an “aging phenotype” over the course of their life spans (Petralia et al., 2014).

While Weismann’s hypotheses were remarkably prescient, at that time neither DNA nor cultured cells were suffisciently understood to allow his theory to be adequately tested. In fact, it was not until nearly one hundred years later, following the development of sophisticated animal cell culture protocols, that he was proven correct: it was shown that somatic cells grown in culture have limited growth potential. After approximately forty passages, human cells stop proliferating and undergo cellular senescence (Hayflick and Moorhead, 1961; Hayflick, 1994). A clear picture has since emerged: somatic cells are mortal, but germ cells are not. Organismal aging begins with a gradual yet consistent decline in cell proliferation of the somatic cells. The macroscopic outcome is evidenced by the appearance of dysfunctional and “worn out” tissues that degrade continuously, finally resulting in structural instability and death of the organism (Weismann, 1891).

**FIGURE 3:**
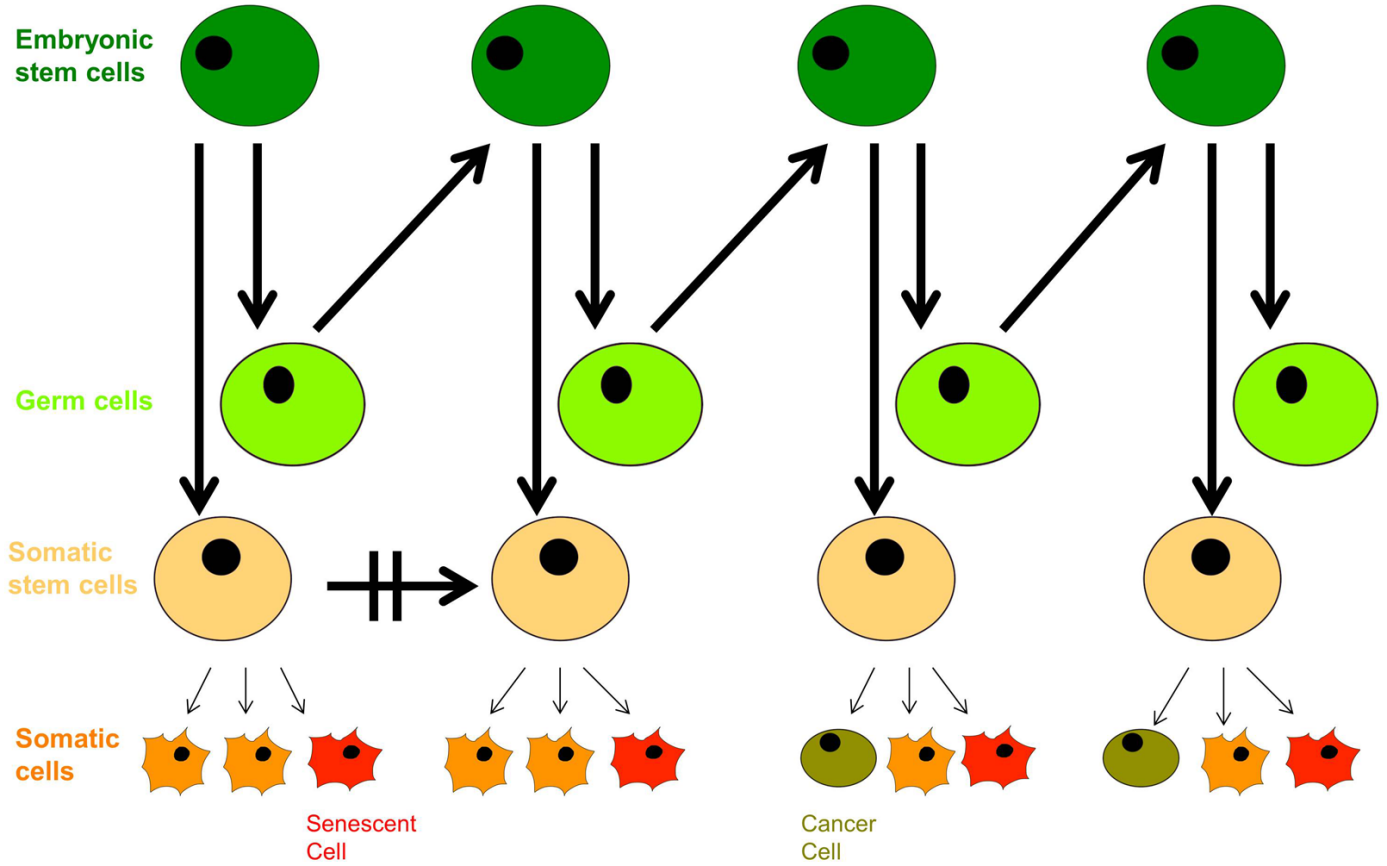
**The dichotomy of germ line and soma: aging and neoplastic transformation.** Totipotent embryonic stem cells give rise to both germ cells and somatic stem cell progenitors with defined self-renewal potential. Somatic progenitors divide and proliferate into tissue-specific terminally differentiated cells with limited self-renewal potential that decreases with age, and they enter senescence in response to external and internal cues. The growing population of senescent cells lead to withered tissue and organismal aging, but deregulation of proliferation arrest as well as secretion of proinflammatory cytokines has pro-oncogenic effects that can trigger neoplastic transformation, evident with the appearance of cancer stem cells; their further aggressive division leads to tumorigenesis.

In the context of modern cell biology, we can extend Weismann’s ideas a little further: recent findings based on cell-fate determination from progenitor cells of variable levels of pluripotency indicate that living organisms are composed of a hierarchy of both limited-capacity somatic and progenitor cells, and high division-capacity stem cells, in addition to immortal germ cells. Emergence of a cancer cell occurs through neoplastic transformation and acquisition of cellular immortality, albeit in a corrupted genetic and epigenetic state, while emergence of an aged cell occurs through the induction of cellular senescence (**Figure 3**). Somatic cells divide *in vivo* for a limited number of cycles, and their cell-division potential is tissue-specific and variable. For instance, intestinal crypt somatic stem cells have greater replicative limits than other types of cells of similar heritage (Potten and Loeﬄer, 1990). Importantly, tissue-specific cell lines replicate with similar potential *in vitro* (Schneider and Mitsui, 1976).

Besides Weismann’s evolutionary theory, many additional theories have been proposed to explain the complexity of aging. These include the antagonistic pleiotropy theory (Williams, 1957), the free radical theory, age-associated shortening of telomeres, development of insulin resistance, decreased immune function, the mitochondrial theory, as well as deregulation of the circadian clock (Campisi, 2013). While these theories indicate functional diversity in the etiology of aging, it must be stressed that each one relies on the concept of internal alterations in individual cells, and does not explain how the microscopic cellular damage manifests as macroscopic aging and tissue breakdown in the organism (with a few exceptions, such as changes in hormone function and declines in immune function). Theories of mutation accumulation and antagonistic pleiotropy address the genetic causes of aging, and environmental stress or lack of it contributes to modulation of the epigenome as well as physiological alterations in different tissues of the whole organism, but each theory revolves around the functional competence of different components of cells and again does not explain how this manifests as macroscopic organismal aging. Experimental evidence unifying the interactions of some components has started to emerge (Kelly, 2011; Sahin et al., 2011), but we propose that *all* of the changes described by diverse theories ultimately *converge* on the cellular senescence theory. However, before we describe this evidence, we will first describe exactly what we mean by cellular senescence.

## The Senescent Phenotype

### Molecular Characteristics of Cellular Senescence

Cellular senescence was first referred to as the “Hayflick limit” based on its discovery in fibroblasts by Dr. Leonard Hayflick (Hayflick, 1965). It has been defined as a process limiting somatic cell proliferation that acts by disrupting cell division, usually entailing loss of replication of nuclear DNA with elevated expression of multiple tumor suppressor proteins (Shay et al., 1991; Rowland et al., 2002). Crucially, and this is important to emphasize, cellular senescence differs from “loss of proliferation” or “quiescence” in that it *also* includes the cellular responses to stress, such as altered gene expression and metabolic activity, as well as accumulated insults that lead to and trigger irreversible arrest. Senescence is additionally characterized by resistance to apoptosis, interference with cellular differentiation, and alteration of cellular function (Moiseeva et al., 2006). Another major hallmark of senescence is the senescence-associated secretory phenotype (SASP), whereby cells convert into their proinflammatory counterparts that promote pathology by secreting various proteins that have deleterious effects on the tissue microenvironment. It is important to realize that while senescence cannot result in loss of proliferation in non-dividing cells such as skeletal muscle cells and neurons, such cells nevertheless undergo a major conversion to the SASP (Salminen et al., 2011).

Since the aforementioned discovery in fibroblasts by Hayflick (1994), we now know that multiple cell types including keratinocytes, endothelial cells, lymphocytes, adrenocortical cells, vascular smooth muscle cells, chondrocytes, and many others acquire senescent phenotypes over time (Rodier and Campisi, 2011). It is an almost universal phenomenon in somatic cells, observed in diverse animal species including mice, chickens, humans and tortoises, and occurs in cells of all ages, from embryonic to adult tissues regardless of donor age (Hayflick, 1994). Nevertheless, some invertebrate animals such as sea urchins appear to have escaped the senescence mechanism, which may be an important area for future research (Francis et al., 2006; Bodnar, 2015).

### Senescence Biomarkers

Morphologically, senescent cells expand in size, acquiring enlarged nuclei, a prominent Golgi apparatus and a vacuolated cytoplasm (Hayflick and Moorhead, 1961). In the absence of DNA replication, metabolically active senescent cells are identified by enlargement of lysosomes accompanied by increased lysosomal β-galactosidase (Kurz et al., 2000; Lee et al., 2006) commonly termed “senescence associated β-galactosidase” (Dimri et al., 1995). In addition to expression of β-galactosidase, most senescent cells also express the tumor suppressor p16^INK4a^, a factor not commonly seen in quiescent or terminally differentiated cells (Alcorta et al., 1996; Hara et al., 1996; Stein et al., 1999). Besides p16^INK4a^, other robust biomarkers of the senescent phenotype are p21/waf1/cip1, p27 and p53 (Shay, 1999). Initiation of autonomous senescence by p16^INK4a^ has also been reported (Dai and Enders, 2000). Senescence-associated heterochromatin foci (SAHF) are another marker that occur in a cell type- and insult-dependent manner and follow expression of p16^INK4a^, but appear to be dispensable for cellular senescence (Kosar et al., 2011). While RNA binding proteins (RBPs) exhibit abundant expression in all tissues and age-groups, their expression level sharply *declines* in senescent cells (Masuda et al., 2012).

The course of senescence is initiated by events such as inhibition of Cdk–cyclin activity through the aforementioned p16^INK4a^ and/or p21, or by alteration of p53 expression that triggers transition from a temporary to a stable cell cycle arrest. Cell-cycle arrest is followed by a state of early senescence marked by lamin B1 down-regulation, marked chromatin remodeling, and SASP production. A true senescent phenotype follows, with the onset of histone proteolysis or replacement and chromatin budding. Finally, the cell terminates in late senescence driven by additional genetic and epigenetic alterations (Shelton et al., 1999; De Cecco et al., 2011; Shah et al., 2013).

### Aging: A Progression of Scale

Once these microscopic events reach a threshold and converge to trigger cellular senescence, the impact then graduates from the subcellular to the tissue level. This picture of aging therefore depends on a progression of scale from small and invisible microscopic damage to larger cellular damage that ultimately results in expression of a highly visible, comprehensively aged phenotype of tissue breakdown throughout the body of the organism. In other words, the *causal nexus* linking the indirect *causes* of aging (microscopic damage) with the direct *effects* of aging (macroscopic) is cellular senescence. And as we move from the microscopic subcellular damage through cellular senescence to macroscopic aging and tissue malfunction, we simultaneously move from causes, to causal nexus, to final effect. It is important to understand that in this model, the visible macroscopic manifestations of aging will *not* become apparent until cells start to senesce, lose their ability to proliferate, and take on the SASP phenotype.

### Senescence as an Anticancer Mechanism

**FIGURE 4:**
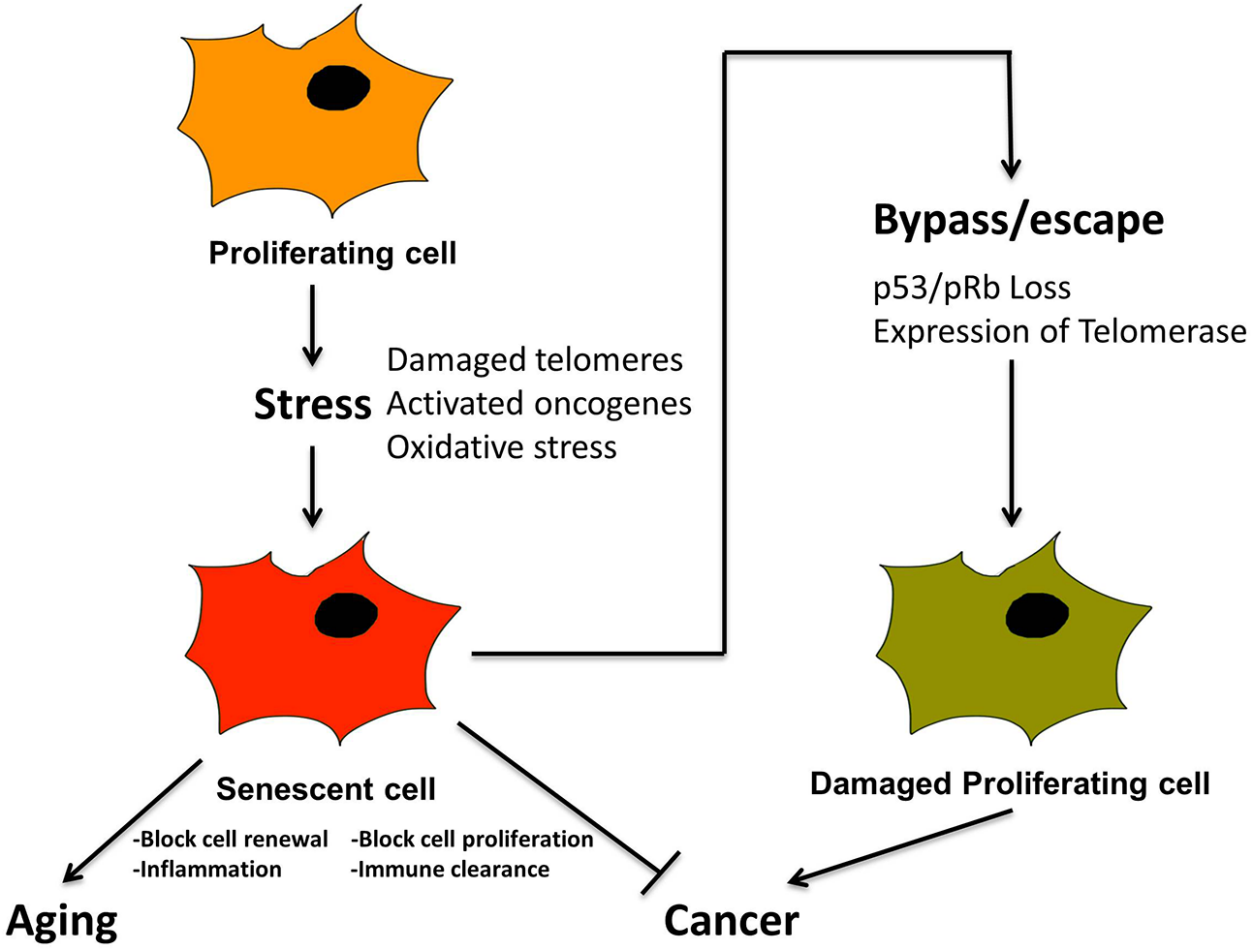
**Senescence is a tumor suppression mechanism, by blocking proliferation of cancer cells, and promotes aging via loss of macroscopic structure and “inflammaging”.** Cancer cells must bypass senescence to continue proliferating. A proliferating cell undergoes stress from, e.g., damaged telomeres and oxidation, leading to activated oncogenes. This leads to cellular senescence, characterized by loss of proliferation, and activation of SASP and inflammation. In combination with immune clearance this is a tumor suppression mechanism. Cancer cells must somehow bypass senescence, i.e., through inactivation of p53 and pRb and/or activation of telomerase, in order to continue proliferating.

Prior to discussing the diverse types of microscopic damage that trigger senescence, it is important to emphasize the relationship between senescence and cancer, since cancer is also a pathological outcome resulting from damaged DNA (Campisi, 2001, 2013; Rodier and Campisi, 2011; Cerella et al., 2015). Cancer is also accompanied by additional specific morphological and molecular features including initially shortened telomeres, unstable chromosomes and, temporarily, relatively low levels of telomerase (Shay and Wright, 2010). Most important conceptually, the onset of cancer is ultimately actualized by cellular dysfunctions resulting from the *bypass* of pathways that would normally induce senescence or apoptosis to prevent cancer (Artandi and Attardi, 2005), (**Figure 4**). For example, inactivation of tumor suppressors such as pRb and P53 and activation of telomerase has been widely detected in many human cancers (**Figure 4**). Such findings have prompted studies to delineate fine differences in pathways leading to apoptosis, cellular senescence and cancer for therapeutic analysis, as well as to decelerate the aging process (Cerella et al., 2015).

## Factors Causing Senescence

**FIGURE 5:**
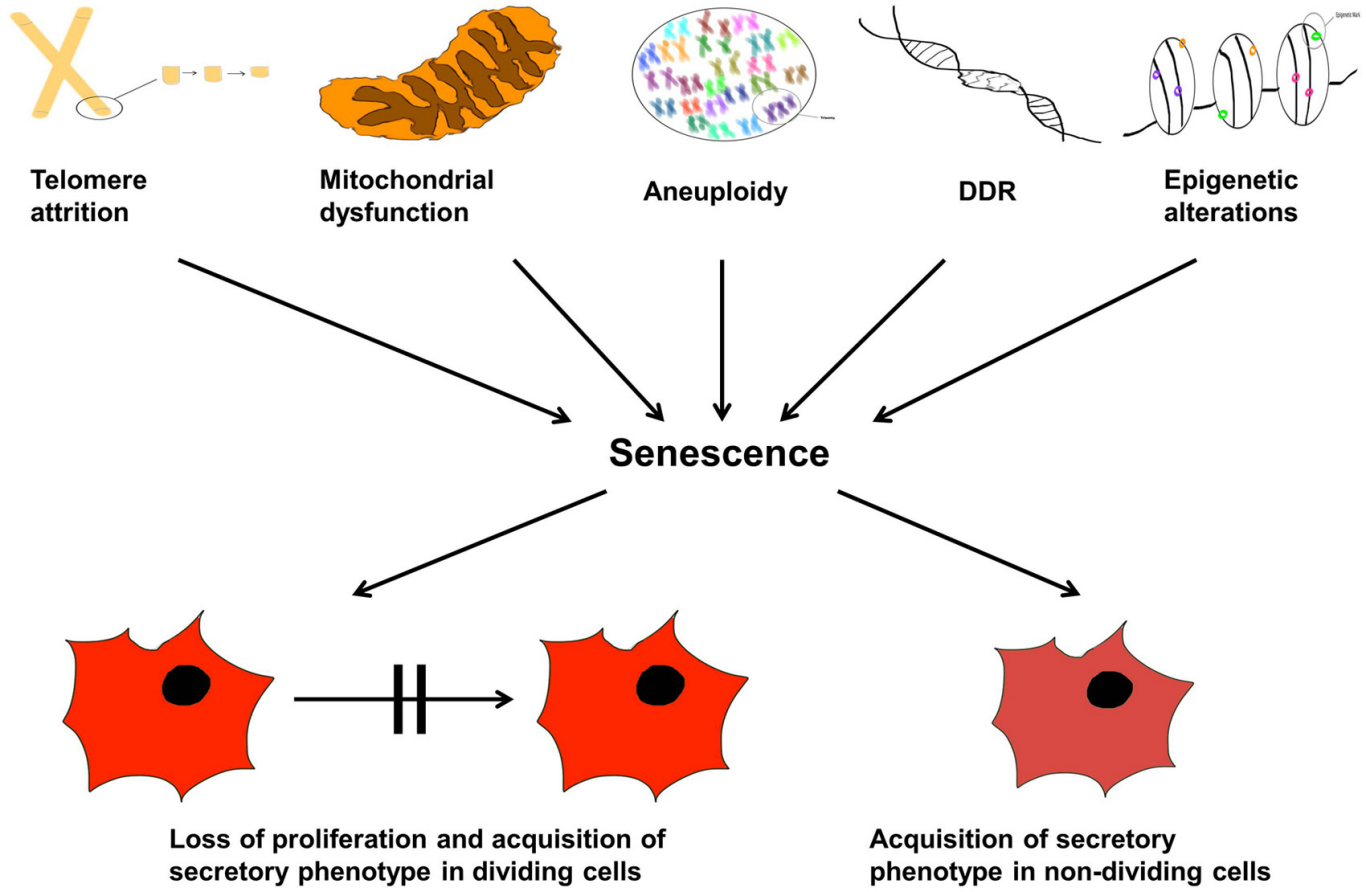
**Cellular senescence and aging.** The organismal life span is controlled by internal and external factors inducing senescence and therefore, proliferation arrest. The text lists molecular and physiological triggers that may lead to: telomere attrition, aneuploidy, DDR response, epigenetic modulations, and mitochondrial dysfunction; these are *causes* of senescence manifested as either loss of cellular proliferation and emergence of the senescent associated secretory phenotype (SASP) in dividing cells, or simply SASP alone in non-dividing cells.

Now that we have described senescence, what are the diverse types of microscopic damage that can cause such a state? They are now known to be multiple (Salama et al., 2014), some of which we describe in the following sections. This is not an exhaustive list, but summarizes some of the key players. This section is also summarized in **Figure 5**.

### Telomere Shortening

Chromosomal structure, as described in this and subsequent sections, is a major determinant of the proliferative ability of cells. Telomeres are probably the most well-studied part thereof because they make a significant contribution to maintaining chromosomal function, and their length is an accurate predicator of the replicative ability of cells (Allsopp et al., 1992). Telomere attrition is associated with senescence (Shay, 1999), and once telomeres reach a critically short length they trigger a DNA damage response, including p53 activation (Herbig et al., 2004; Artandi and Attardi, 2005) that causes onset of cell-cycle arrest and replicative senescence (Kudlow et al., 2008; Gonzalez-Suarez et al., 2009; Benson et al., 2010). In somatic cells of humans and mice, a tight regulation of telomere length and telomerase activity is a significant factor in maintaining balance between induction of senescence and emergence of cancerous cells (Chang, 2005), as well as regulation of life span (Henriques and Ferreira, 2012).

### DNA Double-Strand Breaks and the DNA Damage Response

Besides telomeres, long-term nuclear DNA damage, especially DNA double-strand breaks, leads to a chronic DNA damage response (DDR) signal, that includes activation of a wide range of DDR proteins including p53, leading to senescence (Rodier and Campisi, 2011; Fumagalli et al., 2012). Signals are important for initiation of senescence in response to both short telomeres (see above), and strand breaks, and are manifested as activated DNA damage sensor and effector proteins, localized to either the dysfunctional telomeres (telomere dysfunction-induced foci, TIFs) or other sites of DNA damage such as senescence-associated DNA damage foci or SDFs (d’Adda di Fagagna et al., 2003; Takai et al., 2003; Bartkova et al., 2006; Di Micco et al., 2006). However, *direct* evidence for a causal relationship between DNA breaks and senescence was lacking, due to the difficulty of inducing defined DNA lesions in cells without simultaneously damaging other biomolecules and structures. Recently it was discovered that DNA double-strand breaks (DSBs) alone can create an aged phenotype by implementing an adenovirus-based system based on controlled expression of the Sac I restriction enzyme. The adenovirus was delivered to mice, and molecular and cellular end points in the liver were compared with normally aged animals. Treated mice displayed many signs of normal liver aging as early as 1 month after treatment, including aging pathologies, markers of senescence, fused mitochondria, and alterations in gene expression profiles, showing that DSBs alone can cause distinct aging phenotypes in mouse liver, and proving the role of DNA damage as a driver of tissue aging via induction of senescence (White et al., 2015).

### Epigenetic Alterations

Epigenetic alteration, distinct from telomere, or mutational damage, is another mechanism inducing cellular senescence. Since the only differences between immortal germline cells and mortal somatic cells (**Figure 1**) are changes in gene expression, rather than changes in gene sequences themselves, it stands to reason that a large component of aging is epigenetic in nature. Furthermore, the epigenetic clock is reset or “rejuvenated” during generation of induced pluripotent stem cells (Horvath, 2013).

A collection of recent studies have defined both senescence and youthfulness as being largely epigenetic states (Rando and Chang, 2012; Horvath, 2013; Schellenberg et al., 2014; Beerman and Rossi, 2015; Marioni et al., 2015). Continuous methylation changes at six specific CpG sites of long term cultured cells have been reported (Koch et al., 2012) and in cultured human cells, senescence is accompanied by simultaneous genome-wide DNA hypomethylation in gene-poor, late replicating chromosomal regions and focal low-level hypermethylation at CpG sites of genes that trigger cancer pathways (Cruickshanks et al., 2013). In mouse cells, DNA methylation profiling at CpG islands in promoters of expressed autosomal genes revealed 21% hypermethylation and 14% hypomethylation as a function of age; the data clearly indicate epigenetic dysregulation as a contributing factor for senescence in mammalian cells (Maegawa et al., 2010). Similar age-associated distinct methylation alterations have been detected in the CpG islands of promoters of genes regulating DNA binding and transcription in human brain tissue (Hernandez et al., 2011). Conversely, methylation-based enhanced fidelity of gene transcription has been shown to *promote* longevity (Sen et al., 2015).

Recent work indicates that along with alteration of DNA methylation at specific transcription factor binding sites, nuclear reorganization is also correlated with replicative senescence (Hänzelmann et al., 2015). Noted are extensive nuclear changes, including chromatin remodeling, as integral steps in cellular senescence (Baker and Sedivy, 2013). Relaxation of constitutive heterochromatin and transcription of satellite DNA along with enlargement of centromeres indicate an epigenetic impact on chromosome integrity while perturbation of the epigenome elicits cellular senescence (Berdasco and Esteller, 2012). Also, broad-acting histone-deacetylase inhibitors exert significant epigenetic influence at the onset of senescence (De Cecco et al., 2011).

### Aneuploidy

In addition to DNA damage and epigenetic alterations, aberrant chromosomal number, or aneuploidy also induces senescence (Moiseeva et al., 2006), as does reduced expression of the mitotic checkpoint protein, BubR1. Insufficiency of this protein leads to age-associated pathology and infertility in mice (Baker et al., 2004), whereas increased expression protects against aneuploidy and cancer while extending the lifespan (Baker and Sedivy, 2013; Baker et al., 2013). A p53-mediated mechanism regulates and limits the propagation of aneuploidy in human cells (Thompson and Compton, 2010).

### Oxidative Stress and Mitochondrial Dysfunction

Besides the different types of nuclear alterations described above, mitochondria are also closely linked to senescence. This stands to reason, since insufficient ATP renders the cell incapable of producing sufficient energy to divide and replicate itself. Likewise, oxidative stress generated by mitochondrial dysfunction has been established as a contributing factor in aging (Hayflick and Moorhead, 1961; Simonsen et al., 2002; Moiseeva et al., 2006) as well as an outcome of cellular senescence (Passos et al., 2007).

Most primary cell types are prone to senescence in response to oxidative stress caused by reactive oxygen species (ROS) primarily through telomere attrition (von Zglinicki, 2002), or oncogene activation (Moiseeva et al., 2009). Results from the treatment of human kidney epithelial cells with hydrogen peroxide and the cell-cycle inhibitor apigenin separately showed increased oxidative stress, mitochondrial dysfunction, apoptosis and cellular senescence, and simultaneous treatment augmented the detrimental effects (Small et al., 2012).

A recent review of findings indicates that, in addition to ROS, multiple mitochondrial signaling pathways can induce senescence (Ziegler et al., 2015). For example, reduction in ATP levels by dysregulation of glycolytic enzymes leads to a drastic increase in AMP levels, which induces premature senescence (Zwerschke et al., 2003). Another study showed that a comparison of the global DNA methylation profile of replicative versus premature senescence in lung fibroblasts indicated that oxidative damage partially induces senescence-associated epigenetic alterations (Zhang et al., 2008), linking ROS with epigenetic alterations (see above).

In addition to the physiological processes of mitochondrial dysfunction, damage to mitochondrial DNA (mtDNA) itself also contributes to the aging process through induction and progression of senescence, as evidenced by studies of mouse models with defective mtDNA polymerase that cause accumulation of mutations in mitochondrial DNA due to an impaired proof-reading function (Baines et al., 2014). Damage to mtDNA in these mouse strains leads to the induction of premature aging, evident in progenitor cell malfunction beginning as early as embryogenesis. This results in abnormal lineage differentiation (Ahlqvist et al., 2012). Also, the proliferative ability of mouse embryonic fibroblasts has been confirmed to be altered by a rise in the mutational load in mtDNA (Kukat et al., 2011).

### Inflammation, Energy Sensing, and Altered Metabolic Regulation

Association of chronic inflammation with aging and age-related pathological conditions has long been proposed (Freund et al., 2010). A pro-inflammatory secretory phenotype termed “inflammaging” is associated with senescence in all mammals (Salminen and Kaarniranta, 2012). Along with the stress response, activation of proinflammatory pathways activates NF-κB in the hypothalamus, which in turn negatively influences production of gonadotropin-releasing hormone that normally stimulates neurogenesis in aging mice (Zhang et al., 2008). These findings substantiate evidence of aberrant intercellular communication triggered by secretory properties of senescent cells (the SASP phenotype) responsible for influencing the endocrine system. Protein kinase D1 has been found to regulate RAS-induced oncogenic senescence by modulating NF-κB activity and senescent-associated inflammation (Wang et al., 2014). Interestingly, genetically induced progressive low grade chronic inflammation through knockout of the nfkb1 subunit of transcription factor NF-κB leads to telomere dysfunction, eventually causing senescence (Jurk et al., 2014) and ultimately resulting in early onset of aging (Bernal et al., 2014). A similar mechanism is evident with epidermal growth factor signaling dependent neural stem cell proliferation that is impaired by nitric oxide produced in response to inflammation (Carreira et al., 2014).

Additional pathways that impact cellular senescence through metabolic regulation include the low energy-sensing systems of AMPK and sirtuins (Houtkooper et al., 2010). Sirtuins (SIRT1-7) are an enzyme family that play a significant role in organismal life-span (Mazzoccoli et al., 2014). SIRT1 affects multiple pathways and modulates telomere attrition, inflammation, SASP, and premature senescence (Mercken et al., 2014). SIRT1 participates in maintenance of stem cell pool suppression and regulates the differentiation of progenitor cells (Matsui et al., 2012). Modulation of nutrient and energy-sensing mechanisms is requisite for the regenerative capacity of aging stem cell populations (Mazzoccoli et al., 2014).

### Chronic Mitogenic Signaling

Similar to inflammation, chronic mitogenic signals are another mechanism known to induce cellular senescence (Blagosklonny, 2003). For example, oncogenic induction was first shown to be prompted by a form of H-RAS that chronically triggered a mitogen-activated protein kinase (MAPK) signaling pathway, thereby transforming normal cells into senescent cells (Serrano et al., 1997). Mitogenic signaling and the p16INK4a-Rb pathway cooperate to enforce irreversible cellular senescence. (Takahashi et al., 2006).

### Dysfunctional Extracellular Matrix

Extracellular matrix (ECM) imparts strength and elasticity to normal connective tissue by production of collagen fibrils and other extracellular components by fibroblasts. When damaged, for example in skin, it participates in chronological aging (as well as photoaging from UV and sun exposure) by reducing fibroblast-ECM binding caused by collagen fragmentation (Quan et al., 2013; Fisher et al., 2014). Conversely, injection of cross-linked hyaluronic acid into the skin of aged individuals stimulated fibroblasts, and increased synthesis of Type I collagen that helped to enhance mechanical force. This was evident in the elongation and spreading of cells and indicates that decline of mechanical support by the ECM contributes to the onset of cellular senescence (Quan et al., 2013).

Now that we have described some of the diverse types of subcellular damage (causes) that can trigger senescence, what is the evidence that senescence itself causes an aged phenotype (effect)?

## Evidence that Cellular Senescence is the Causal Nexus of Aging

Various sources of evidence support the connection between cellular senescence (loss of proliferation and SASP) and the induction of organismal aging.

### Correlation Between *In Vitro* Lifespan and Species Lifespan

The replicative lifespans of mammalian fibroblasts *in vitro* were studied in a number of cell cultures representing eight species. Senescence occurred at population doubling levels that were characteristic for the species, and was shown to be related to the species’ maximal lifespan by a strict power law, being proportional to the square root of the maximal lifespan. In this regard, “Based on data in the literature, the same relationship was also valid for the lifespans of circulating mammalian erythrocytes *in vivo*. These results may indicate the prevalence of a common functional basis regulating the lifespan of fibroblasts and erythrocytes and thus operating in replicative as well as postmitotic cells *in vitro* and *in vivo*” (Rohme, 1981). However, it should also be noted that a later study of similar mammalian species found that the replicative lifespan of fibroblasts correlated with the body mass of the species rather than the longevity, which admittedly could be a factor confounding the correct interpretation of the data (Lorenzini et al., 2005). Furthermore, the authors were critical of the conclusions of the earlier work (Rohme, 1981) because of the inclusion of embryonic and adult fibroblasts.

### Progeroid Syndromes are Characterized by Premature Senescence

The role of cellular senescence as the cause of aging is supported by examining diseases of premature aging such as Hutchinson-Gilford progeria syndrome (HGPS) and Werner’s syndrome (WS) (von Zglinicki et al., 1995; Zindy et al., 1997; Saha et al., 2013). Specific causative genes have been identified in both of these syndromes. In humans, cells taken from patients with both of these progeroid syndromes undergo far fewer divisions than normal cells (Salk et al., 1981). Cellular senescence is likely the key mechanism underlying the pathological conditions of premature aging in both syndromes (Dominguez-Gerpe and Araujo-Vilar, 2008). In human HGPS, cells exhibit a senescent phenotype including accumulation of Progerin that leads to nuclear and mitotic abnormalities; the mouse model of HGPS shows a relatively weaker effect (Varga et al., 2006). Admittedly, such findings could challenge the implication of pathways participating in aging through arrest of cell proliferation and onset of senescence (Miller, 2004), but it could be that Progerin simply does not induce cellular senescence strongly in mouse cells.

### Senescent Cells Accumulate *In Vivo* in Aging Organisms

Age-associated accumulation of senescent cells in various tissues and organs causes functional disruption of tissue structure, partly through appearance and accumulation of senescent-associated secretory proteins (Coppé et al., 2008; Rodier and Campisi, 2011). Aging effects have been detected in cells from multiple mammalian species of various ages, including mice, baboons and humans (Herbig et al., 2006; Jeyapalan et al., 2007; Wang et al., 2009). A heterogeneous cell population showing a variable proportion of senescent cells interspersed in islands of normal cells is a common feature of aging mammalian tissues (Bahar et al., 2006; Herbig et al., 2006). Variability in populations of senescent cells in tissues can be explained by the fact that onset of senescence is triggered by diverse factors that lead to accumulation of molecular and physiological damage in cells along with the exhaustion of their replicative potential (Shawi and Autexier, 2008). Based on the tissue-specific variability of such factors throughout the lifespan of the organism and the extent of exposure to different stressors in the habitat/environment of the organism, a variable population of senescent cells in tissue is a classic and characteristic feature of the progressive nature of aging (Shawi and Autexier, 2008). *In vitro*, a negative correlation between donor age and proliferative lifespan has been proposed (Hayflick, 1965; Allsopp et al., 1992). Nevertheless, there are also reports indicating that despite using controlled health status and culture conditions, replicative lifespan in cultured fibroblasts does not correlate with donor age (Cristofalo et al., 1998).

### Restoration of Telomeres and Cell Proliferation Reverses the Aged Phenotype

Restoration of telomerase activity can prevent replication-dependent telomere attrition *in vitro* (Vaziri and Benchimol, 1998). Expression of exogenous human telomerase reverse transcriptase (hTERT) has been achieved in a variety of cells (Bodnar et al., 1998) and is known to counteract replicative senescence by extending the proliferative lifespan (Simonsen et al., 2002). This can circumvent the telomere-dependent DNA damage signal and avoid induction of senescence (d’Adda di Fagagna et al., 2003). Remarkably, similar reactivation of telomerase *in vivo* in late generations of genetically engineered telomerase-deficient transgenic mouse strains (TERT-ER) resulted in the extension of typically short telomeres and reversed neurodegeneration and other degenerative features in the spleen, intestine, and testes (Jaskelioff et al., 2011). Similarly, TA-65, a small molecule activator of telomerase, decreased the percentage of critically short telomeres and increased telomere length and lifespan in mice, without increasing cancer incidence (Bernardes de Jesus et al., 2011). These findings reinforce the significance of telomerase activity in maintaining chromosomal integrity, while also demonstrating that declining activity represents an important factor contributing to the onset of senescence and aging.

### Removal of Senescent Cells Reverses Aging

Instead of reversing senescence, as described above, actual removal or “deletion” of senescent cells through genetic manipulation in mice has been shown to yield reversal of age-related symptoms in a tissue-specific manner, and to attenuate the progression of established age-related symptoms when such cell-clearance was induced later in the lifespan (Baker et al., 2011). Similarly, “Senolytic” drugs that specifically kill senescent cells by targeting pro-survival networks and making them susceptible to apoptosis were observed to extend healthspan in mice (Zhu et al., 2015).

### Inhibition of Cell Proliferation Directly Causes an Aged Phenotype

Recently, two crucial, seminal discoveries provide evidence that aging can be induced in the absence of any molecular damage, indicating that it is the senescent phenotype itself with its inherent cessation of cellular proliferation and SASP that causes aging, rather than biochemical or other intrinsic cues. This is suggested by the recent and remarkable observations that *in vivo*, tissue and organismal aging can be artificially forced to occur in the absence of *any* accumulated molecular damage in only two steps: (1) proliferative arrest followed by (2) organismal aging (Pruitt et al., 2013; Boquoi et al., 2015).

Induced expression of p16I^NK4a^ in newborn and just-weaned mice of transgenic strains with competence for conditional p16^INK4a^ expression revealed inhibition of proliferation and accelerated emergence of aging features, including weight loss and loss of subcutaneous fat, hair loss, wrinkled skin morphology, and cataracts (Boquoi et al., 2015). These symptoms were abrogated in the mutant background of Cdk4 R24C, since this strain is insensitive to kinase inhibitors. These data confirm that conditional p16^INK4a^ expression results from Cdk inhibition (Boquoi et al., 2015).

Likewise, conditional induction of the Cdk inhibitor Cdkn1b or p27kip1 caused either complete loss or significant decline of somatic cell proliferation in multiple tissues and induced premature aging features in the absence of any detectable naturally senescent cells in aged tissue (as opposed to the artificially blocked cellular proliferation) or activation of any DDR pathways (Pruitt et al., 2013).

### Caloric Restriction Decreases Senescent Cells

Caloric restriction (CR) is one of the few demonstrably effective modalities shown to be capable of slowing down aging in almost all species. But how is this accomplished? It is likely that it works at least in part by suppressing cellular senescence. For example, temporary dietary restriction in middle-aged mice has been successful in decreasing the number of senescent cells in the liver and intestine (Wang et al., 2009, 2010). Markers of cellular oxidative damage decrease in cells in which senescence was noted, as well as in calorically restricted mice (Kirkland, 2010).

Species-specificity of rodents in response to CR has been shown in controlled laboratory conditions, where CR increased the median life-span of rats by 14–15%. In mice the finding was much weaker, ranging from 4 to 27%, with inbred strains showing the least effect in comparison to outbred strains, indicative of genotype dependence (Swindell, 2012). Experimental evidence further indicates that CR is more effective in males, as well as in standard model organisms used for experiments than in non-model organisms. These findings are indicative of convergent adaptation in laboratory conditions (Nakagawa et al., 2012).

### Viruses and Drugs that Exhaust Cell Proliferation are Associated with an Aging Phenotype

The existence of cancer has been evident in fossil records prior to emergence of *homo sapiens* on the earth (Dunn, 2012). Multiple co-morbidities due to cancer, and particularly its chemotherapeutic treatment, are augmented in aging cancer survivors, who have a rate of frailty akin to older adults (Avis and Deimling, 2008; Ness et al., 2013; Henderson et al., 2014). Notably, anticancer drug treatment induces apoptosis in hair follicles leading to hair loss in treated patients through senescence associated p53 mediated pathways (Botchkarev et al., 2000). Similarly, a blockade of cell-proliferation by the alopecia-causing chemotherapeutic drug cyclophosphamide is evident: it down-regulates Sonic Hedgehog (Shh) transcription, thereby affecting the Shh-dependent proliferation pathway in actively proliferating epithelial cells (Xie et al., 2015). Likewise, the mechanism of action of most chemotherapeutics is such a blockage of cell division, which has a pro-aging effect.

It is highly likely that infection with retroviruses such as HIV and the side-effects of antiretroviral drugs accelerate the senescence of cells, which in turn leads to premature aging. Notably, age-associated co-morbidities due to diabetes, cardiovascular conditions, frailty, and neurocognitive impairment are more prevalent in HIV-infected individuals of all ages than those without infection (Pathai et al., 2014). For example, a comparative analysis on the brains of HIV-infected and non-infected controls using Magnetic Resonance Imaging and a series of neuropsychological tests revealed acceleration of the normal aging trajectory in higher-order cognition regions of brains of infected individuals, despite absence of any detectable symptoms of dementia (Pfefferbaum et al., 2014). It has been postulated that a comparative analysis of HIV-associated lipodystrophy with genetic lipodystrophy as well as those associated with monogenic laminopathies might reveal the shared pathways leading to disorders of adipose distribution and the effects of drugs and therapeutic approaches used to treat infection (Freitas and Carvalho, 2013). Based on increasing evidence of premature aging after successful HIV treatment, analysis of the mechanism of nucleoside analog anti-retroviral drugs revealed progressive accumulation of somatic mitochondrial mutations akin to the accumulation evident in the late stage of life as a result of normal aging (Payne and Chinnery, 2015). Also, a comparative analysis of methylation levels of peripheral blood mononuclear cells in age-matched HIV-1 infected and uninfected individuals revealed that HIV-1 infection accelerated age-related methylation by 13–14 years, thereby indicating that HIV-1 associated aging shares the same functional and epigenetic changes associated with normal aging (Rickabaugh et al., 2015).

The findings cited indicate that pathological conditions and infections as well as their therapeutic interventions contribute to senescence regardless of biological age.

## Summary and Conclusion

Aging is a complex, multifaceted, and multidimensional state, with a variable course of progression. This work represents a compilation of the most recent findings in support of Weismann’s original hypothesis that the initial decline and cessation of the proliferative ability of fatigued, damaged, and dysfunctional cells is the ultimate cause of aging. Following the principle of cause and effect, our model demonstrates how replicative senescence, first referred to as the Hayflick response, allows several competing aging theories to coexist and converge. At the same time, it encompasses Weismann’s predictions of complementary mortal (soma) and immortal (germ) cell types to account for a capacity for infinite renewal juxtaposed against mortality. By integrating the wisdom of many great minds, we propose a slightly evolved model that not only accurately describes the data, but also enables a complete understanding of the interactions between the mechanisms at play over the course of aging.

**FIGURE 6:**
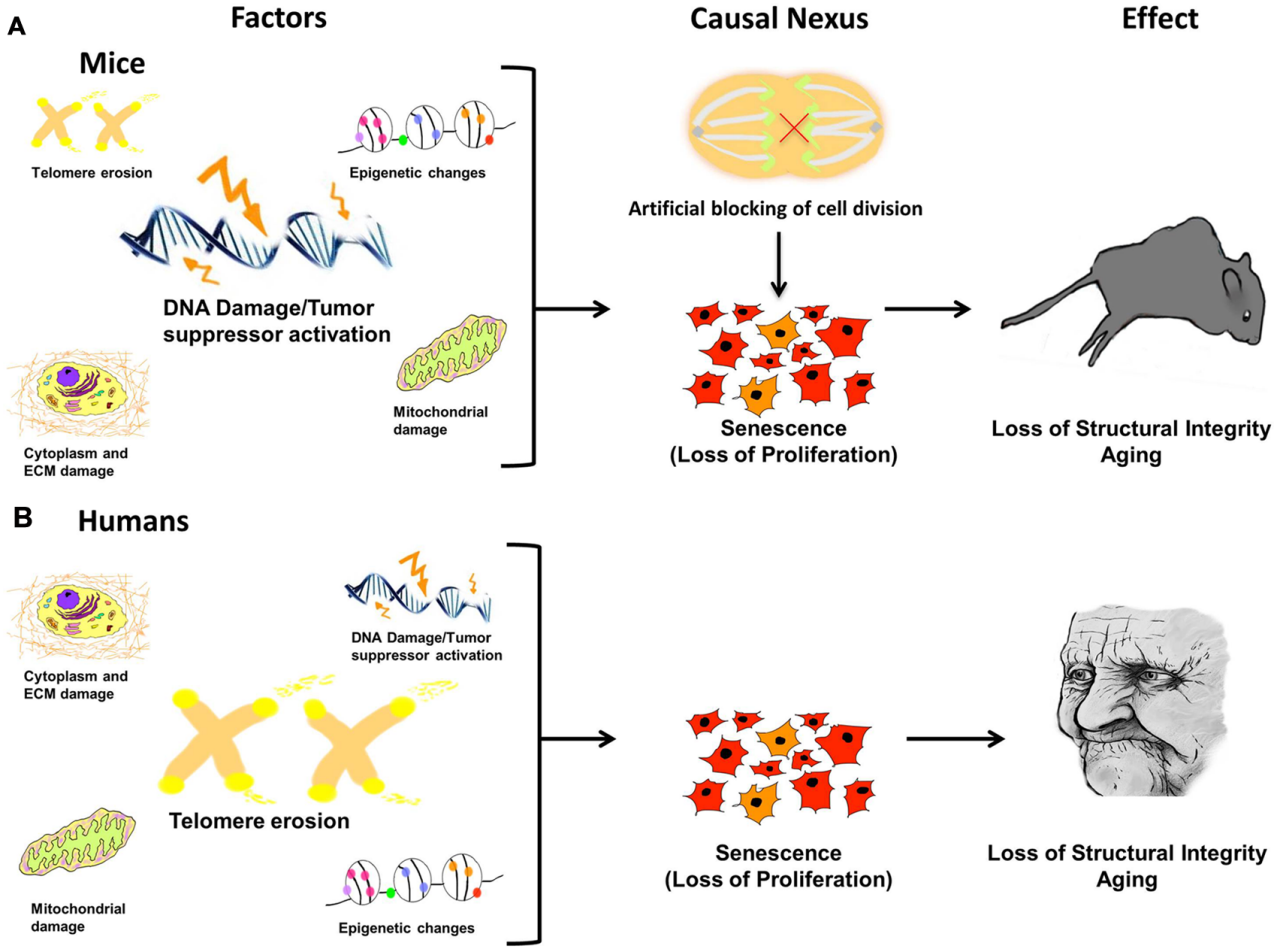
**Damage leads to causal nexus leads to effects.** In different species the causes of cellular senescence may be similar, but have different magnitudes. For example, in mice **(A)** DNA damage/Tumor suppressor activation may be a major factor, whereas in humans **(B)**, telomere erosion may have a greater effect because humans have much shorter telomeres than mice. In both species, accrued, and unrepaired damage to the cellular genome, epigenome, and organelles (causes) is finally manifested in the visible phenotype, evident as aged features of the organism, such as organ dysfunction, loss of structural integrity, and physiological and anatomical changes (effect). The link between cause and effect, the *causal nexus*, is cellular senescence. Additionally, it has been shown that in mice, blocking cell division alone, in the absence of any molecular damage, can induce premature Progeria-like aging.

Since aging is a progressive condition that steadily advances from invisible to visible and localized to ubiquitous, the central question as to the direct cause of the entire process is key. The answer has been elusive due to its complex nature. Our model proposes that the process of aging results from a sequential passage through three distinct phases and can be described by the following blueprint: (1) molecular damage which results in (2) cessation of proliferation leading to cellular senescence followed by (3) body-wide aging of the organism (**Figure 6**). The first step occurs when localized, microscopic damage accumulates to a point where the burden to repair overwhelms the system. Despite the tissue source or broad input of molecular damage, crossing of this threshold results in the second phase, the crux of the entire process – arrest of cellular proliferation, acquisition of the SASP, and imminent cellular senescence. Once this occurs, the third phase of aging begins. This final phase is marked by tissue dysfunction and breakdown that results in the visible signs of comprehensive organismal aging.

In different species, the causes of cellular senescence may be similar, but have different magnitudes (**Figure 6**). This may help explain why aging studies in different species are not always comparable and thus can be difficult to interpret. For example, in mice (**Figure 6A**) DNA damage/tumor suppressor activation may be a major factor, whereas in humans (**Figure 6B**), telomere erosion may have a greater effect because humans have much shorter telomeres than mice. Nevertheless, in both species, accrued and unrepaired damage to the cellular genome, epigenome, and organelles (causes) is finally manifested in the visible phenotype, evident as aged features of the organism, such as organ dysfunction, loss of structural integrity, and physiological and anatomical changes (effect). Therefore, the link between cause and effect, the causal nexus, is cellular senescence.

The incremental advance proposed by our model is that while there are many undisputed factors that trigger the onset of cellular senescence and result in cessation of proliferation and SASP, the first phase in the model (cumulative molecular damage) is a *precursor*, rather than a final cause of aging. The complexity normally imposed by countless variables (i.e., age of onset, site of damage, affected cell type, mechanism of damage, and even species) that need to be overcome is rendered manageable by eliminating the first phase in the aging schematic. And since organismal aging can be artificially and reversibly induced by blocking and restarting cellular proliferation, this indicates that the second phase in the model – cessation of proliferation followed by cellular senescence – clearly represents the *essential* cause of aging. Placing cellular senescence in the pivotal junction between cause and effect, the causal nexus, to yield an integrated model of aging will serve to advance identification of crucial targets for future therapeutic investigation. By identifying cellular senescence as the causal nexus of aging, the process of treating, reversing and possibly even eventually eliminating this once inevitable outcome draws closer to reality.

## Author Contributions

The authors confirm being the sole contributors to this work and approved it for publication.

## Conflict of Interest Statement

The authors declare that the research was conducted in the absence of any commercial or financial relationships that could be construed as a potential conflict of interest. The reviewer FBJ and handling Editor declared their shared affiliation, and the handling Editor states that the process nevertheless met the standards of a fair and objective review.
